# SG-APSIC1117: Healthcare-associated infections among the obstetrics and gynecology patients with confirmed COVID-19 in Hung Vuong Hospital, Vietnam

**DOI:** 10.1017/ash.2023.21

**Published:** 2023-03-16

**Authors:** Ngo Nhung, Hang Tran, Nhung Ngo, Anh Dinh, Nga Nguyen, Tham Ngo, Thang Vu, Duy Nguyen, Hang Phan

**Affiliations:** Obstetrics and Gynaecology, Ho Chi Minh City, Vietnam; Infection Control Department, Hung Vuong Hospital, Ho Chi Minh City, Vietnam; Infection Control Department, Hung Vuong Hospital, Ho Chi Minh City, Vietnam; Infection Control Department, Hung Vuong Hospital, Ho Chi Minh City, Vietnam; Infection Control Department, Hung Vuong Hospital, Ho Chi Minh City, Vietnam; Infection Control Department, Hung Vuong Hospital, Ho Chi Minh City, Vietnam; Infection Control Department, Hung Vuong Hospital, Ho Chi Minh City, Vietnam; Infection Control Department, Hung Vuong Hospital, Ho Chi Minh City, Vietnam; Vice President, Hung Vuong Hospital, Ho Chi Minh City, Vietnam

## Abstract

**Objectives:** During the COVID-19 surge, our hospital was overloaded due to the increasingly high volume of patients and lack of resources, which resulted in difficulties in complying with infection control and prevention (IPC) practices. In this study, we estimated healthcare-associated infection (HAI) incidence and relevant factors among COVID-19 patients in Hung Vuong hospital. **Methods:** This study included all SARS-CoV-2–positive adult patients hospitalized between September 1 and October 31, 2021. The Centers for Disease Control and Prevention definition of HAI in the acute-care setting was used. **Results:** Among 773 patients, 21 (2.72%) developed 26 separate HAIs. The cumulative days of hospitalization were 5,607. The incidence of HAI among COVID-19 patients was 4.64 per 1,000 days of hospitalization. The most frequent HAI was clinically defined pneumonia (46.2%), for which the ventilator-associated pneumonia (VAP) rate was 41.9 per 1,000 ventilator days. Among 21 positive cultures, the most frequently isolated microorganisms were *pseudomonas aeruginosa, Klebsiella pneumoniae*, and *escherichia coli*. HAIs were significantly associated with the number of central-line days (OR, 1.74; 95% CI, 1.33–2.78), the number of indwelling urinary catheter days (OR, 1.46; 95% CI, 1.05–2.03), the length of administration days (OR, 1.25; 95% CI, 1.07–1.45), antibiotics use prior to HAIs (OR, 0.01; 95% CI, 0.01–0.21), and the number of nasal cannula days (OR, 0.62; 95% CI, 0.44–0.85). **Conclusions:** COVID-19 makes patients more vulnerable and may require more invasive procedures, increasing the infection risk by opportunistic pathogens like gram-negative Enterobacteriaceae. Hence, fundamental IPC recommendations should be strongly implemented.

